# One Step Further in the Characterization of Synthetic Polymers by Ion Mobility Mass Spectrometry: Evaluating the Contribution of End-groups

**DOI:** 10.3390/polym11040688

**Published:** 2019-04-16

**Authors:** Quentin Duez, Romain Liénard, Sébastien Moins, Vincent Lemaur, Olivier Coulembier, Jérôme Cornil, Pascal Gerbaux, Julien De Winter

**Affiliations:** 1 Organic Synthesis and Mass Spectrometry Laboratory, Interdisciplinary Center for Mass Spectrometry (CISMa), University of Mons, UMONS, 23 Place du Parc, 7000 Mons, Belgium; quentin.duez@umons.ac.be (Q.D.); romain.lienard@umons.ac.be (R.L.); pascal.gerbaux@umons.ac.be (P.G.); 2 Laboratory for Chemistry of Novel Materials, Center of Innovation and Research in Materials and Polymers (CIRMAP), University of Mons, UMONS, 23 Place du Parc, 7000 Mons, Belgium; vincent.lemaur@umons.ac.be (V.L.); jerome.cornil@umons.ac.be (J.C.); 3 Laboratory of Polymeric and Composite Materials, Center of Innovation and Research in Materials and Polymers (CIRMAP), University of Mons, UMONS, 23 Place du Parc, 7000 Mons, Belgium; sebastien.moins@umons.ac.be (S.M.); olivier.coulembier@umons.ac.be (O.C.)

**Keywords:** mass spectrometry, ion mobility, polymers, data fitting, ion structure

## Abstract

Several families of polymers possessing various end-groups are characterized by ion mobility mass spectrometry (IMMS). A significant contribution of the end-groups to the ion collision cross section (CCS) is observed, although their role is neglected in current fitting models described in literature. Comparing polymers prepared from different synthetic procedures might thus, be misleading with the current theoretical treatments. We show that this issue is alleviated by comparing the CCS of various polymer ions (polyesters and polyethers) as a function of the number of atoms in the macroion instead of the usual representation involving the degree of polymerization. Finally, we extract the atom number density from the spectra which gives us the possibility to evaluate the compaction of polymer ions, and by extension to discern isomeric polymers.

## 1. Introduction

Due to its successful coupling to Mass Spectrometry (MS), Ion Mobility Spectrometry (IMS) has become increasingly popular for probing the shape of gaseous ions [1]. This technique allows separation of ions as a function of their mobilities in a buffer gas under the influence of an electric field. The ion drift time across the mobility cell is directly proportional to their collision cross-section (CCS), which strictly corresponds to a momentum transfer cross-section. However, it is often considered in first approximation to reflect the 3D structures of gaseous ions [2,3,4]. While the concept of Ion Mobility Mass Spectrometry (IMMS) dates back to the 1960s, its versatility and recent commercialization fueled fundamental studies of a large range of analytes, such as biomolecules, host-guest systems, and synthetic polymers [5,6,7,8,9,10,11]. 

In the field of polymers, IMMS is mostly used to follow the evolution of the CCS of various macroions as a function of their charge states and degree of polymerization (DP) to ultimately make it a powerful analytical tool. In this context, many polymers have been considered, such as poly(ethylene glycol) (PEG) [12,13], poly(propylene glycol) (PPG) [14,15,16], poly(ethylene terephthalate) (PET) [17], poly(styrene) (PS) [18], poly(methyl methacrylate) (PMMA) [15], polylactide (PLA) [9,19], and poly(ε-caprolactone) (PCL) [20]. Although linear polymers were mainly investigated, it is worth stressing that more complex structures were also considered, such as cyclic or star-shaped macromolecules [20,21,22,23]. Due to the intrinsic dispersity of polymers, it is possible to follow the evolution of CCS for a large range of chain lengths by analyzing a limited number of samples. Recently, some groups went a step further by deriving physicochemical data and structural interpretations by fitting CCS/DP evolutions. For instance, Kokubo and Vana introduced a model based on globular structures allowing determination of the characteristic ratio and dielectric constant of polyethers [24,25]. Haler et al. reported a method to deduce the structure of gaseous polymer ions without any input from computational chemistry [26]. Both works used a power law with an (0,0) intercept to rationalize CCS as a function of DP evolutions in the form of $CCS=A\;DP^{\frac{2}{3}}$; forcing the (0,0) intercept thus implies that any following analysis is made by neglecting the contribution of end groups. The power exponent was constrained to 2/3 to describe the evolution of spherical objects and the parameter A was left free [9,27]. 

In the present contribution, we aim to probe the contribution of the various polymer end-groups typically encountered in polymerization strategies to the CCS chain-size evolution. To avoid possible misleading interpretations arising from literature data, we will, thus, synthesize polymers with various chain ends and the measured CCS will be plotted against the DP. We will also modify the reported methodologies by restraining the type of ions that should be considered for fitting to only the spherical macroions [9,27]. Each data set will be fitted by the equation $CCS=A\;DP^{\frac{2}{3}}+B$, with both the A and the B parameters being fitting parameters. In a second approach, another representation of the CCS evolutions taking into account the size, in terms of number atoms, of the monomer units as well as the size of the end groups will be introduced to characterize globular polymer ions as a function of their compaction.

## 2. Materials and Methods

Polylactides with M_n_ = 2000 and 4000 g mol^−1^, poly(ε-caprolactones) with M_n_ = 3000 and 4000 g mol^−1^, and polypropiolactones with M_n_= 2000 and 4000 g mol^−1^ were freshly synthetized by using the procedures described below. All other compounds (α-methyl, ω-hydroxy-poly(ethylene oxide)s (PEO) with M_n_ = 750, 1150, 2000 g mol^−1^, *N*,*N*′-dicyclohexylcarbodiimide (DCC), 4-dimethylaminopyridine (DMAP), 1,8-Diazabicyclo[5.4.0]undec-7-ene (DBU), benzoic acid, 2-benzoylbenzoic acid, 3,3,3-triphenylpropionic acid, hexanoic acid, and palmitic acid were purchased from Sigma Aldrich and used without further purification, except DBU, which was dried over CaH_2_ before being distilled. Trifluoromethane sulfonic acid (TfOH) was purchased from Merck and used as received. Propiolactone is supplied by VWR and was dried on CaH_2_ for 24 h and distillated under vacuum. Methanol used as an initiator was dried on molecular sieves 3 Å for 48 h. CHCl_3_ and CH_2_Cl_2_ solvent was dried using a MBraun Solvent Purification System (model MB-SPS 800) equipped with alumina drying columns.

Preparation of α-methyl, ω-hydroxy-poly(L-lactide) (PLA) for M_n_ = 2000 g mol^−1^: In a glovebox under nitrogen pressure (O_2_ < 5 ppm, H_2_O < 1 ppm), a vial was charged with L-lactide (L-LA) (1.00 g, 6.9 mmol). CH_2_Cl_2_ (10.0 g) was added, followed by the addition of methanol (30 µL, 0.74 mmol) and DBU (112 µL, 0.74 mmol). After 1 min under stirring, benzoic acid (100 mg, 0.8 mmol) was added. The as-obtained DBU/benzoic acid salt and residual L-LA were removed by precipitation into cold methanol to give α-methyl, ω-hydroxy-P(L-LA). The amounts of methanol (10 µL, 0.25 mmol) and DBU (39 µL, 0.26 mmol) and the polymerization time (2 min 30 s) were varied to produce PLA M_n_ = 4000 g mol^−1^.

Preparation of α-benzyl, ω-hydroxy-poly(ε-caprolactone) (PCL) for M_n_ = 1700 g mol^−1^: In a glovebox under nitrogen atmosphere (O_2_ < 5 ppm, H_2_O < 1 ppm), a glass vial was charged with 320 µL ε-caprolactone (ε-CL) (2.8 mmol), 16.9 mg benzoic acid (0.14 mmol), and 12 µL benzyl alcohol (0.11 mmol). The mixture was let to stir at 155 °C for 20 h and was subsequently cooled down and diluted in a minimum amount of CH_2_Cl_2_. After solubilization, the medium was precipitated in cold heptane to give α-benzyl, ω-hydroxy-PCL.

Preparation of α-methyl, ω-hydroxy-poly(propiolactone) (PPL) for expected M_n_ = 1800 g mol^−1^: In a previously flame-dried vial, PL (187 mg, 2.9 mmol) and CH_3_OH (5 µL, 0.12 mmol) were solubilized in 3.5 g of CHCl_3_. After 10 min under stirring at 50 °C, a solution of TfOH in CHCl_3_ (11 µl of TfOH (0.12 mmol) in 0.49 g of CHCl_3_) was injected into the medium ([PL]_0_ = 0.71 M, [PL]_0_/[CH_3_OH]_0_/[TfOH]_0_ = 24/1/1). The polymerization was conducted at 50 °C for 5 min, cooled down, and precipitated twice in cold heptane.

General procedure for chain end functionalization: To start, 100 mg of polymer (PEO or PLA) (1 equivalent) were dissolved in 3 mL of tetrahydrofuran (THF). To the solution of polymer, 3 equivalents of carboxylic acid-based reagents (benzoic acid, 2-benzoylbenzoic acid, 3,3,3-triphenylpropionic acid, hexanoic acid, or palmitic acid), and 1 equivalent of *N*,*N*′-dicyclohexylcarbodiimide (DCC) and 4-dimethylaminopyridine (DMAP) were then added. After 24 h under stirring at room temperature, the polymers were recovered via precipitation into cold diethyl ether (for PEOs) or cold hexane (for PLAs), dried under vacuum, and used as is.

**Figure 1:**
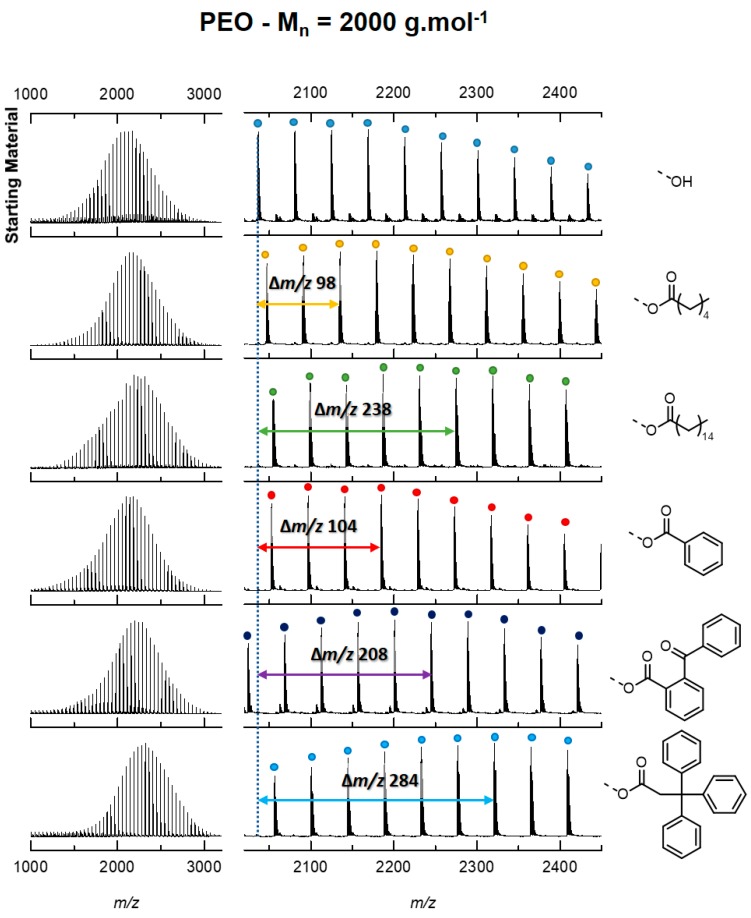
MALDI mass spectra recorded for functionalized and non-functionalized PEO with M_n_ = 2000 g mol^−1^. Functionalization was attested by the appearance of additional signals with adequate mass differences compared to the α-methyl, ω-hydroxy PEO used as a starting material (top).

End-capped polymer characterization: In the present paper, we did not optimize the end-capping procedures, since using mass spectrometry, mass discrimination between end-capped and pristine macromolecules is readily achieved. Each functionalized polymer sample was analyzed by mass spectrometry (MS) using a Matrix-assisted Laser Desorption/Ionization (MALDI) ion source. Partial or quantitative functionalization was attested by the appearance of additional signals with adequate mass differences compared to the precursor α-methyl, ω-hydroxy polymers. For the sake of illustration, Figure 1 presents the MALDI mass spectra recorded for functionalized and non-functionalized PEOs with M_n_ = 2000 g mol^−1^.

Ion Mobility Mass Spectrometry: Ion mobility mass spectrometry experiments were conducted with a Waters Synapt G2-S*i* (Manchester, UK), a Traveling Wave Ion Mobility instrument equipped with an Electrospray (ESI) ion source. Polymer solutions were prepared in acetonitrile at a final concentration of 0.5 µM and NaI was added to reach a concentration of 15 µM in Na^+^. These solutions were infused in the ESI ion source with a flow rate of 5 µL min^−1^ using a Harvard Pump11 Elite (Holliston, MA, USA) syringe pump. ESI parameters used (Positive ion mode): Capillary voltage at 3.1 kV, sampling cone at 30 V, source temperature at 100 °C, and desolvation temperature at 200 °C. Ion mobility experiments were performed using N_2_ as a drift gas and a wave height of 40 V. Experiments were carried out three times with varied wave velocity (400–800 m s^−1^) and drift gas flow (60–90 mL min^−1^). Arrival times were extracted using Waters MassLynx and subsequently converted to collision cross sections (CCS) in helium by the polymer calibration using poly(ethylene glycol) and PLA as calibrants [28]. Reported CCS values are averaged over three sets of experiments and calibrations. It should be emphasized that the conversion from N_2_ arrival times to He CCS is susceptible to induce bias depending on the ion’s nature, charge state, and size [3,29]. However, since we analyse here ions having similar chemical natures, sizes, and charge states to our calibrants, we do believe that the possible errors are negligible and do not alter the conclusions drawn in the present manuscript.

**Figure 2:**
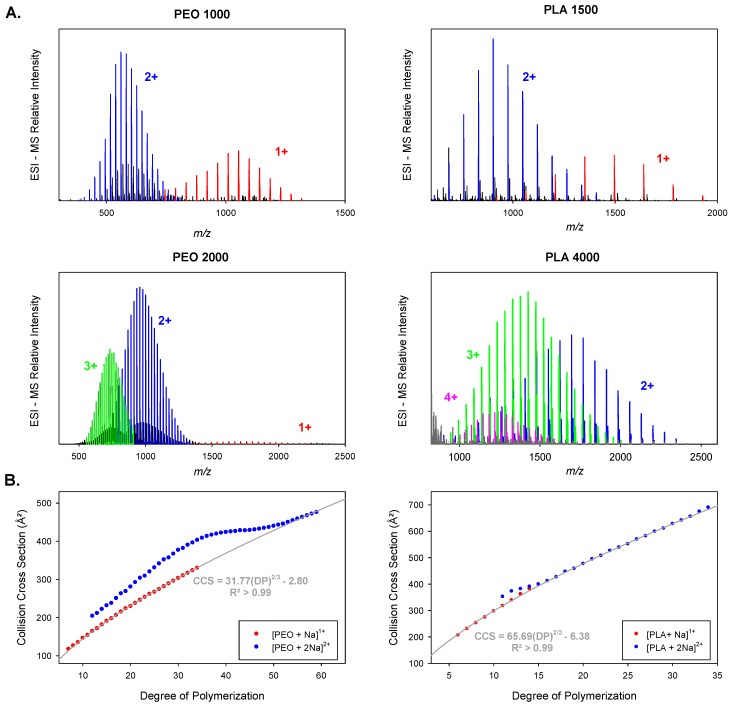
(**A**) Electrospray (ESI) Mass spectra of α-methyl, ω-hydroxy-Poly(ethylene oxide) (PEO) 1000, poly(L-lactide) (PLA) 1500, PEO 2000, and PLA 4000. Detected ion series with different charge states, 1+, 2+, 3+, and 4+ are highlighted in different colors. (**B**) Collision cross section evolution of singly and doubly charged PEO and PLA ions as a function of the degree of polymerization (DP). Fittings of the data corresponding to globular structures (singly charged and doubly charged beyond the plateau) are shown in dark grey along with fitting equations and regression coefficients.

Data treatment and fitting: Data processing and fittings were performed using Sigmaplot 11.0 (Systat, San Jose, CA, USA). All data points used for fittings are shown in each figure, except for Figure 2.

## 3. Results

### 3.1. Model Description

Upon ESI, α-methyl, ω-hydroxy-PEO and PLA polymers are detected as adducts with sodium ions (Figure 2A). For simplicity, each PX polymer (X = EO or LA) with a M_n_ = 1000 g mol^−1^ will be named PX 1000 in the rest of this manuscript. Polymer ions are subjected to ion mobility experiments and are separated according to their electrophoretic mobilities in the buffer gas [1,3]. From such measurements, the ion drift times through the mobility cell can be recorded and converted into collision cross sections (CCS). In the current literature, ion mobility data are often represented for a given charge state as a function of the degree of polymerization (DP) or as a function of *m*/*z* to account for the evolution of the ion shapes along the polymer distribution [9,10,20,21,30]. CCS are here recorded for singly and doubly charged PEO and PLA and plotted as a function of DP (Figure 2B). To keep the discussion focused, higher charge states will not be involved in the fitting analysis.

Whereas the CCS of singly charged oligomers increases steadily with growing DP, the CCS of doubly charged oligomers presents a non-monotonic evolution. This behavior is extensively described in the literature and is associated with different conformations for gaseous polymer ions [9,12,19,30]. Singly charged polymer ions fold around the cation to form a globule, while small multiply charged ions adopt stretched shapes due to the Coulombic repulsion of the complexed cations and are often described as “beads on a string” conformations [30]. Longer chains can screen the electrostatic repulsion and fold into globular species. The tipping point between stretched and globular conformations is characterized by a plateau in the CCS/DP or CCS/*m*/*z* evolution [9,12,19,30]. Here, we only fit the ion mobility data for the globular species, for which the constrained 2/3 power law is well established. We also use the $CCS=A\;DP^{\frac{2}{3}}+B$ equation, where *A* and *B* are free parameters, so that the possible contribution of the chain ends to the CCS is not neglected [24,25,26]. To strengthen the postulate that chain ends should not be ignored when fitting ion mobility data, α-methyl ω-hydroxyl PEO and PLA samples are functionalized on their hydroxy end groups by molecules of growing sizes, containing either aliphatic or aromatic groups (See Figure 1 and Table 1). 

### 3.2. Contribution of the Chain Ends to the Collision Cross Section

The α-methyl ω-hydroxy PEO and PLA samples were esterified using five aliphatic and aromatic acids. The naming convention of the produced α-methyl ω-ester polymers is presented in Table 1. MALDI mass spectra of each functionalized sample are shown in Figure 1 for PEO2000 and all others are reported in Figures S1–S4.

**Figure 3:**
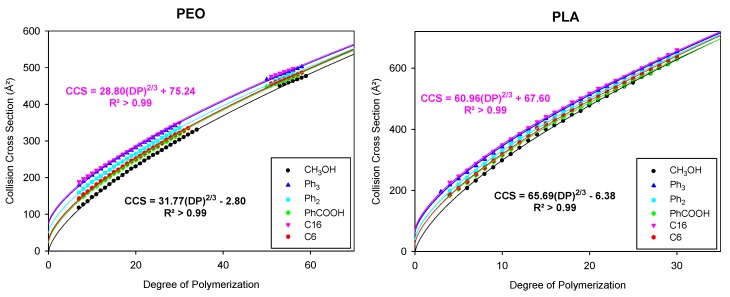
Collision cross section evolution as a function of the degree of polymerization (DP) for singly and doubly charged globular ions for α-methyl ω-hydroxy and α-methyl ω-ester-PEO and PLA samples. Fittings are performed using the equation $CCS=A\;DP^{\frac{2}{3}}+B;$ the equations and regression coefficients for the smallest (CH_3_OH) and bulkiest (C16) functionalized polymers are shown.

Ion Mobility Mass Spectrometry (IMMS) analyses were performed on the pristine and functionalized PEO and PLA. The evolution of the CCS as a function of DP for globular singly and doubly charged ions are represented in Figure 3. We recall here that we consider only the globular structures for the fits, whereas the full range of CCS/DP values are plotted in Figure 2 (compact and extended ions).

We observe that the CCS evolution is specific for each polymer, thus demonstrating that the size of the chain ends should not be ignored when their contribution to the global CCS is not negligible, especially for small chains and bulky end-groups. Each data set is successfully fitted by the equation $CCS=A\;DP^{\frac{2}{3}}+B$, with regression coefficients consistently larger than 0.99. The parameters *A* and *B* obtained for each polymer are reported in Table 2. The *B* parameter appears to increase with the size of the chain end and has similar values for a given end group irrespective of the polymer backbone nature. The small deviations between PEO and PLA, as well as the fact that *B* is slightly negative for methanol-initiated polymers, are attributed to fitting and CCS calibration uncertainties. It should be emphasized that *B* does not represent the exact CCS of the cationized end groups but rather stands for the contribution of the chain end to the global CCS. Indeed, our model considers globular ions as perfect spheres. However, depending on the location of the bulky chain ends in the globular ion, either on the surface or within the core of the spherical object, they might contribute differently to the global CCS. The *A* parameters are consistent among the polymer backbones, irrespective of the end group, with a maximum deviation less than 10%. From the *A* parameters, it appears that the CCS of PLA increases much more per added monomer unit than for PEO, which is not surprising given that a lactidyl unit contains three times more atoms than an ethylene oxide unit. 

Thus, it appears that the *B* parameter of the fitting for CCS/DP evolutions provides information on the contribution of the chain end to the global CCS. Although the information of the nature of the monomer unit is contained in the *A* parameter, this is also slightly impacted by the end-groups. To provide a more general method for the cross-comparisons of polymers irrespective of the nature of their monomer units or chain ends, we suggest representing the CCS evolutions as a function of the total number of atoms.

### 3.3. Considering Different Chain Ends and Polymer Backbones by Counting Atoms

As introduced by Bowers et al. when describing the evolution of the CCS of various polyethers with different monomer sizes [14], the representation of CCS as a function of the number of atoms (*nAtoms*) helps accounting for the size of the chain ends and of the monomer units. In Figures S5 and S6, we separately plot the evolution of the CCS as a function of the atom numbers for PEO and PLA bearing the different end-groups. Each polymer data set may be fitted by a single curve described by $CCS=A^{\prime }nAtoms^{\frac{2}{3}}$. In this type of representation, the equation encompasses the (0,0) intercept, since the CCS is nullified when there is no atom. *A*′ includes information about the contribution of each additional atom, and by extension of each additional monomer unit, to the global CCS. Indeed, as presented in Table 3, the *A*′ factor seems to be clearly associated with the nature of the monomer unit, since for the PEO and PLA polymers, the averaged *A*′ factors respectively amount to 8.55 ± 0.07 and 9.29 ± 0.03. The weak differences between the *A*′ parameters for polymers presenting different side chains arises from the fact that when considering the contribution of the end groups only in terms of the number of atoms, we implicitly assume that the chemical nature of the corresponding atoms does not impact the CCS evolution. In other words, we assume that the end groups do not participate in the growing of the sphere. Actually, the weak influence of the chain-end nature to the *A*′ factor comes from the following aspects. In the present systems, we are dealing with globular or spherical macro ions (singly charged ions or doubly charged ions after the folding), with the charged site being entangled somewhere within the globule and being associated with the monomer unit carbonyl groups, as described in our previous papers using Molecular Dynamics simulation [9,19]. The chain ends that are not involved in the charge solvation, are thus more than likely located at the outer surface of the sphere, making some interactions with the surface groups. This indicates that the structures of the selected end groups are not likely to play a significant role in the shaping of the polymer molecules towards the overall chain conformation. In future works, we will consider polymers with charged bulky end-groups or end-groups that participate in the charge stabilization, as those end-groups will be more than likely responsible for the folding of the gaseous polymer ions.

**Figure 4:**
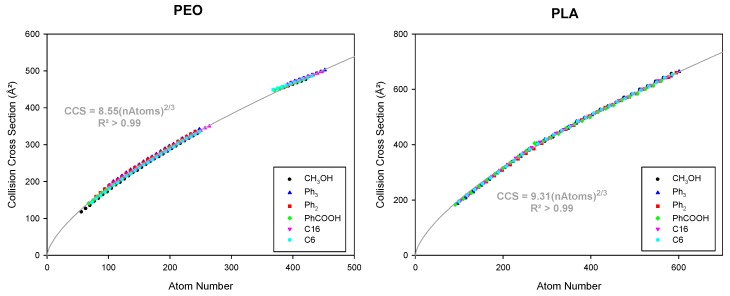
Evolution of the collision cross section as a function of the number of atoms for singly and doubly charged globular ions of pristine and functionalized PEO and PLA. Fittings were performed using the equation $CCS=A^{\prime }\;nAtoms^{\frac{2}{3}};$ the equations and regression coefficients of the two fits are shown.

For the present data set, the impact of the structure of the chain ends on the *A*′ parameter remains weak, as confirmed by Figure 4, in which we combined the CCS data of all the PEO and PLA polymers as a function of the total atom numbers and by only considering the chain end contribution in terms of atom numbers. All data points of a given type of polymer align on one unique curve (regardless of the nature of the end-groups), meaning that the sole number of atoms in these end-groups seems to be a good estimate for their contribution, with no consideration of the chemical nature of these atoms. 

From the global data of Figure 4, we derive a parameter *A*′ directly related to the nature of the polymers. As the influence of the chain end chemical nature to the *A*′ parameter is negligible here, as discussed above, the CCS evolutions of any polymer become a characteristic of the polymer backbone.

**Figure 5:**
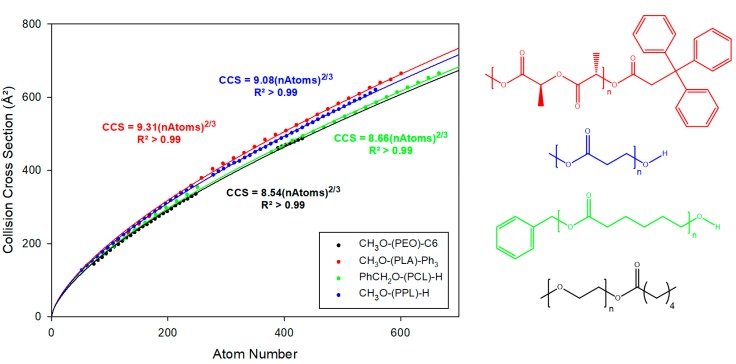
Evolution of the collision cross-section as a function of the number of atoms for singly and doubly charged globular ions produced from α-methyl ω-C6-PEO (Black), α-methyl ω-Ph_3_ -PLA (Red), α-methyl ω-hydroxy-PPL (Blue), and α-benzyl ω-hydroxy-PCL (Green). Fittings are performed using the equation $CCS=A^{\prime }\;nAtoms^{\frac{2}{3}};$ the equations and regression coefficients of each fit are also reported.

To reinforce this observation, IMMS analyses were performed on polymers with various backbone natures and end groups, namely α-methyl ω-C6-PEO, α-methyl ω-Ph_3_-PLA, α-methyl ω-hydroxy-poly(propiolactone) (PPL), and α-benzyl ω-hydroxy-poly(ε-caprolactone) (PCL). Recorded drift times are converted to CCS and are represented in Figure 5 for each globular object as a function of the number of atoms in the macroion.

Interestingly, the CCS evolutions for each polymer backbone are rationalized by a different equation. Each additional monomer unit, therefore, contributes differently to the global CCS regardless of the chain end. By assuming that the CCS of a globular ion corresponds to the projection surface of a perfect sphere, i.e., a disk, the sphere volume can be derived from the CCS by Equation (1). This assumption is clearly harsh, as the CCS does not strictly correspond to a surface but to a momentum transfer cross-section, as ion mobility spectrometry does not directly probe surfaces but ion mobilities [4]. We still make this approximation as we aim here to compare the compaction of globular polymer backbones and not to derive exact physicochemical values. Additionally, based on the fitting curve, we can also determine the CCS by Equation (2). Finally, by combining both equations, it is possible to determine the atomic number density, Equation (3). The value is multiplied by 1000 to have the atomic number density by nm^3^ instead of in Å^3^.

(1) $$V=\;\frac{4}{3}\sqrt{\frac{CCS³}{\pi }}$$

(2) $$CCS=A\prime \;{\left(n_{atoms}\right)}^{2/3}$$

(3) $$\frac{n_{atoms}}{V}\left[nm^{-3}\right]=\frac{3\sqrt{\pi }}{4\sqrt{A\prime^{3}}}\;1000$$

**Figure 6:**
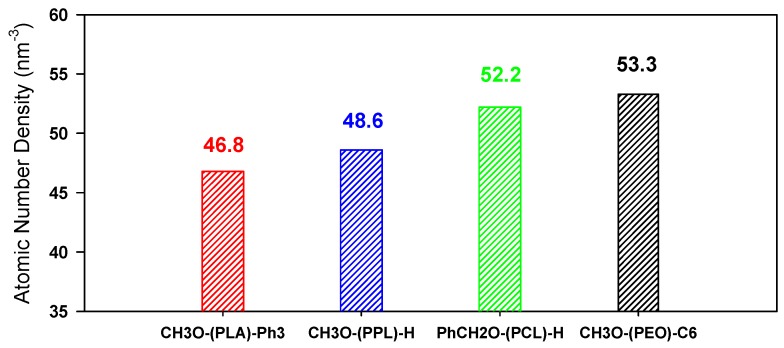
Atomic number densities determined by using Equation (3) for the different polymers.

Therefore, by using the *A*′ parameter from each fitting presented in Figure 5, an atomic number density can subsequently be determined for each polymer family, as illustrated in Figure 6. 

Interestingly, it is now possible to classify folded polymers ions based on the atomic number density independently of the end-groups. PLA and PPL backbones are isomeric, as each lactidyl unit is the isomer of two consecutive propiolactone units. Since the presence of the pending CH_3_ groups in the lactidyl units hinders the compaction of the structure, the contribution of each monomer unit to the global CCS is, thus, expected to be larger for PLA than for PPL, in full consistency with Figure 6. In addition, for polyesters, it appears that increasing the number of methylene groups induces a better compaction of the structure due to the enhanced flexibility of the polymer chain. Finally, PEO folds into more compact globules for the same number of atoms due to the lack of carbonyl moiety in the monomer unit. It should be emphasized that small deviations in atomic number densities will likely be observed for different end groups, as discussed above for deviations in the *A*′ parameters. However, these divergences will be negligible compared to the compaction of various polymer backbones.

Depending on the type of representation, whether by reporting ion mobility data as a function of DP or number of atoms, the contribution of chain ends to the global CCS or the contribution of each additional atom (and monomer unit) to the global CCS can, thus, be highlighted but are never neglected.

## 4. Conclusions

In the present paper, we discussed IMMS data fitting methodologies reported in the current literature with the aim of deriving physicochemical data and structural interpretations, while neglecting the contribution of chain ends to the global CCS. Due to the diversity of synthetic strategies leading to the possibility to initiate end-cap polymer backbones by different end groups, these procedures are found to promote ambiguous interpretations. This issue can be solved with an adapted approach, also considering the contribution of the chain ends to the CCS. To highlight this, polymers with various chain ends were synthetized and analyzed by IMMS. Only globular ions were considered since, to the best of our knowledge, they are the only type of ions for which a mathematical model describing the size evolution of their CCS exists and is validated among the IMMS community. It appears that the CCS evolutions can only be rationalized by the equation $CCS=A\;DP^{\frac{2}{3}}+B$, where B corresponds to the contribution of the chain ends to the global CCS. However, this type of equation makes cross-comparison of different polymer backbones unreliable, since the size of each monomer unit is not considered. In contrast, representation of IMMS data as a function of the number of atoms accounts for both the size of end groups and monomer units. Atomic number densities can be further derived for four polymer families to describe the relative compaction of globular macroions. When isomeric polylactide and poly(propiolactone) chains are considered, it appears that the presence of the pending methyl groups in polylactide is responsible for the lower atomic number density. 

We hope that this work will raise awareness among the polymer mass spectrometry community that fitting laws for ion mobility data should be as universal as possible and should consider the diversity of possible end groups. We also hope that this work will fuel further fundamental studies on gaseous polymer ions.

## Figures and Tables

**Table 1:** Naming convention for the functionalized polymers.

End-Groups	Naming	Number of Atoms *
CH_3_O/H	CH_3_OH	6
CH_3_O/C(=O)C_5_H_11_	C6	23
CH_3_O/C(=O)C_15_H_31_	C16	53
CH_3_O/C(=O)Ph	PhCOOH	18
CH_3_O/C(=O)Ph(C=O)Ph	Ph_2_	30
CH_3_O/C(=O)CH_2_CPh_3_	Ph_3_	44

Note: * The number of atoms of the end-groups are reported regardless of the nature (H, C, O) of the atoms. (Ph stands for Phenyl).

**Table 2:** Collision cross section evolution as a function of the degree of polymerization—parameters obtained from fitted curves for a $CCS=A\;DP^{\frac{2}{3}}+B$ equation (see also Figure 3).

	PEO		PLA	
End-Groups	A	B	A	B
CH_3_O/H	31.77	−2.80	65.69	−6.38
CH_3_O/C(=O)C_5_H_11_	30.77	27.41	63.90	22.93
CH_3_O/C(=O)C_15_H_31_	28.80	75.24	60.96	67.60
CH_3_O/C(=O)Ph	30.91	22.65	62.85	24.00
CH_3_O/C(=O)Ph(C=O)Ph	29.63	49.12	62.78	38.13
CH_3_O/C(=O)CH_2_CPh_3_	28.89	70.88	61.21	60.77

**Table 3:** Collision cross section evolution as a function of the atom numbers—parameters obtained from fitted curves for a $CCS=A^{\prime }\;nAtoms^{\frac{2}{3}}\;$ equation (See Figures S5 and S6).

		PEO	PLA
End-Groups	Atom Number in the End-Groups *	A′	A′
CH_3_O/H	6	8.42	9.26
CH_3_O/C(=O)Ph	18	8.55	9.28
CH_3_O/C(=O)C_5_H_11_	23	8.54	9.31
CH_3_O/C(=O)Ph(C=O)Ph	30	8.62	9.26
CH_3_O/C(=O)CH_2_CPh_3_	44	8.63	9.31
CH_3_O/C(=O)C_15_H_31_	53	8.55	9.33
Average		8.55 ± 0.07	9.29 ± 0.03

* The numbers of atoms of the end-groups are reported regardless of the nature (H, C, O) of the atoms. (Ph stands for Phenyl).

## Supplementary Materials

The following are available online at https://www.mdpi.com/2073-4360/11/4/688/s1. Figure S1. MALDI mass spectra recorded for functionalized and non-functionalized PEO with Mn = 750 g mol^−1^. Functionalization was attested by apparition of additional signals with adequate mass differences compared to the α-methyl, ω-hydroxy polymer. Figure S2. MALDI mass spectra recorded for functionalized and non-functionalized PEO with Mn = 1150 g mol^−1^. Functionalization was attested by apparition of additional signals with adequate mass differences compared to the α-methyl, ω-hydroxy polymer. Figure S3. MALDI mass spectra recorded for functionalized and non-functionalized PLA with Mn = 2000 g mol^−1^. Functionalization was attested by apparition of additional signals with adequate mass differences compared to the α-methyl, ω-hydroxy polymer. Figure S4. MALDI mass spectra recorded for functionalized and non-functionalized PLA with Mn = 4000 g mol^−1^. Functionalization was attested by apparition of additional signals with adequate mass differences compared to the α-methyl, ω-hydroxy polymer. Figure S5. Evolution of the collision cross section as a function of the number of atoms for singly and doubly charged globular ions for pristine and functionalized PEO and PLA with aliphatic end groups. Fittings were performed using the equation $CCS=A^{\prime }\;nAtoms^{\frac{2}{3}};$ the equations and regression coefficients of each fit are shown. Figure S6. Evolution of the collision cross section as a function of the number of atoms for singly and doubly charged globular ions for functionalized PEO and PLA with aromatic end groups. Fittings were performed using the equation $CCS=A^{\prime }\;nAtoms^{\frac{2}{3}};$ the equations and regression coefficients of each fit are shown.
