# Supplementary material for: One Step Further in the Characterization of Synthetic Polymers by Ion Mobility Mass Spectrometry: Evaluating the Contribution of End-groups
Source: Polymers (Basel). 2019 Apr 16;11(4):688. doi: 10.3390/polym11040688 (PMC6523221; doi:10.3390/polym11040688)
Supplement: Supplementary file 1 [file polymers-11-00688-s001.pdf]

One step further in the characterization of synthetic polymers by ion mobility mass spectrometry: Evaluating the contribution of end-groups

Quentin Duez [1,2], Romain Liénard [1,3], Sébastien Moins [3], Vincent Lemaur [2], Olivier Coulembier [3], Jérôme Cornil [2], Pascal Gerbaux [1] and Julien De Winter [1,*]

[1] Organic Synthesis and Mass Spectrometry Laboratory, Interdisciplinary Center for Mass Spectrometry (CISMa)

[2] Laboratory for Chemistry of Novel Materials, Center of Innovation and Research in Materials and Polymers (CIRMAP)

[3] Laboratory of Polymeric and Composite Materials, Center of Innovation and Research in Materials and Polymers (CIRMAP)
University of Mons, UMONS, 23 Place du Parc, 7000 Mons, Belgium

* Correspondence: julien.dewinter@umons.ac.be

Supplementary Information

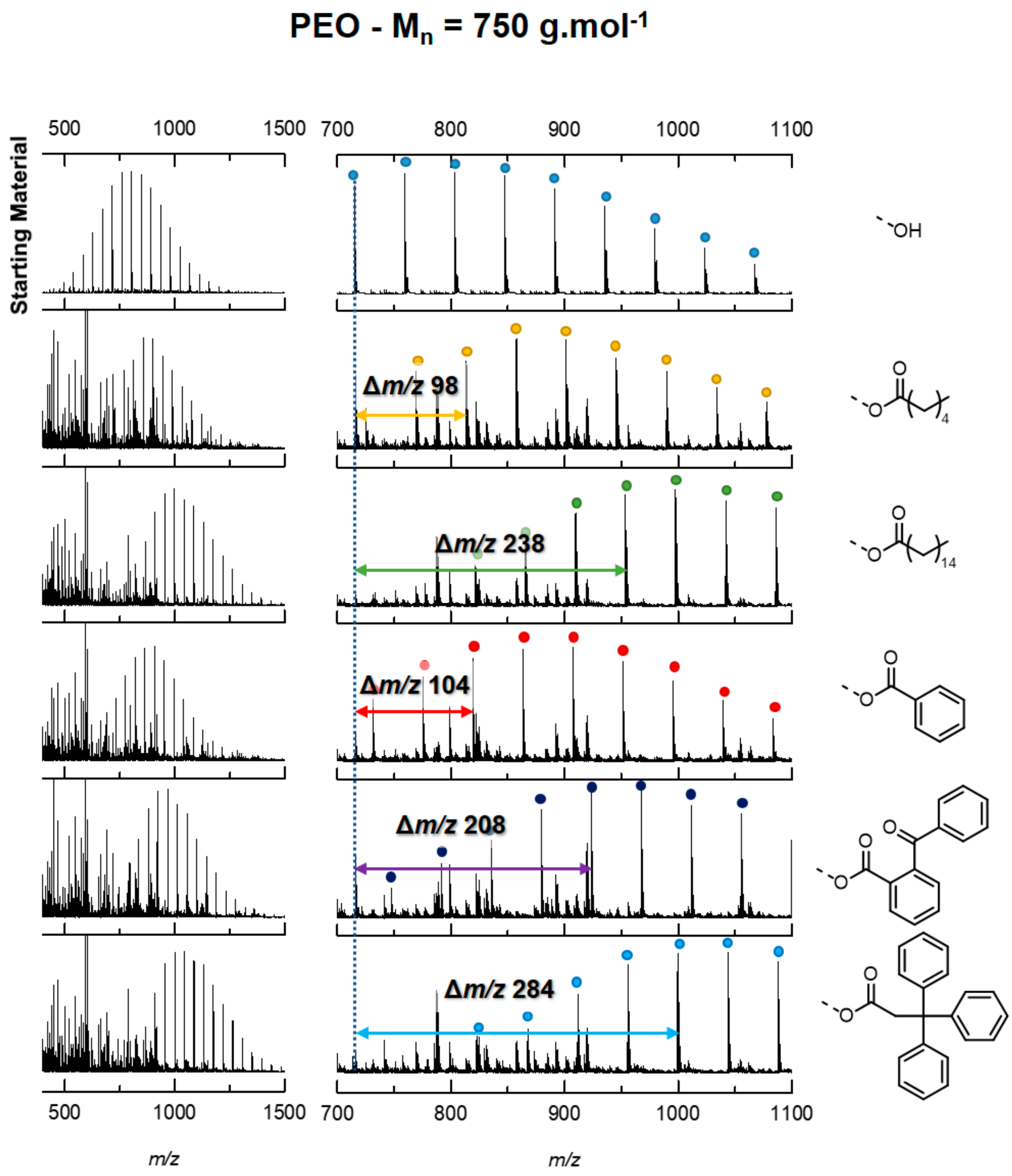

Figure SI1. MALDI mass spectra recorded for functionalized and non-functionalized PEO with M_n = 750 g mol^{-1}. Functionalization was attested by apparition of additional signals with adequate mass differences compared to the α-methyl, ω-hydroxy polymer.

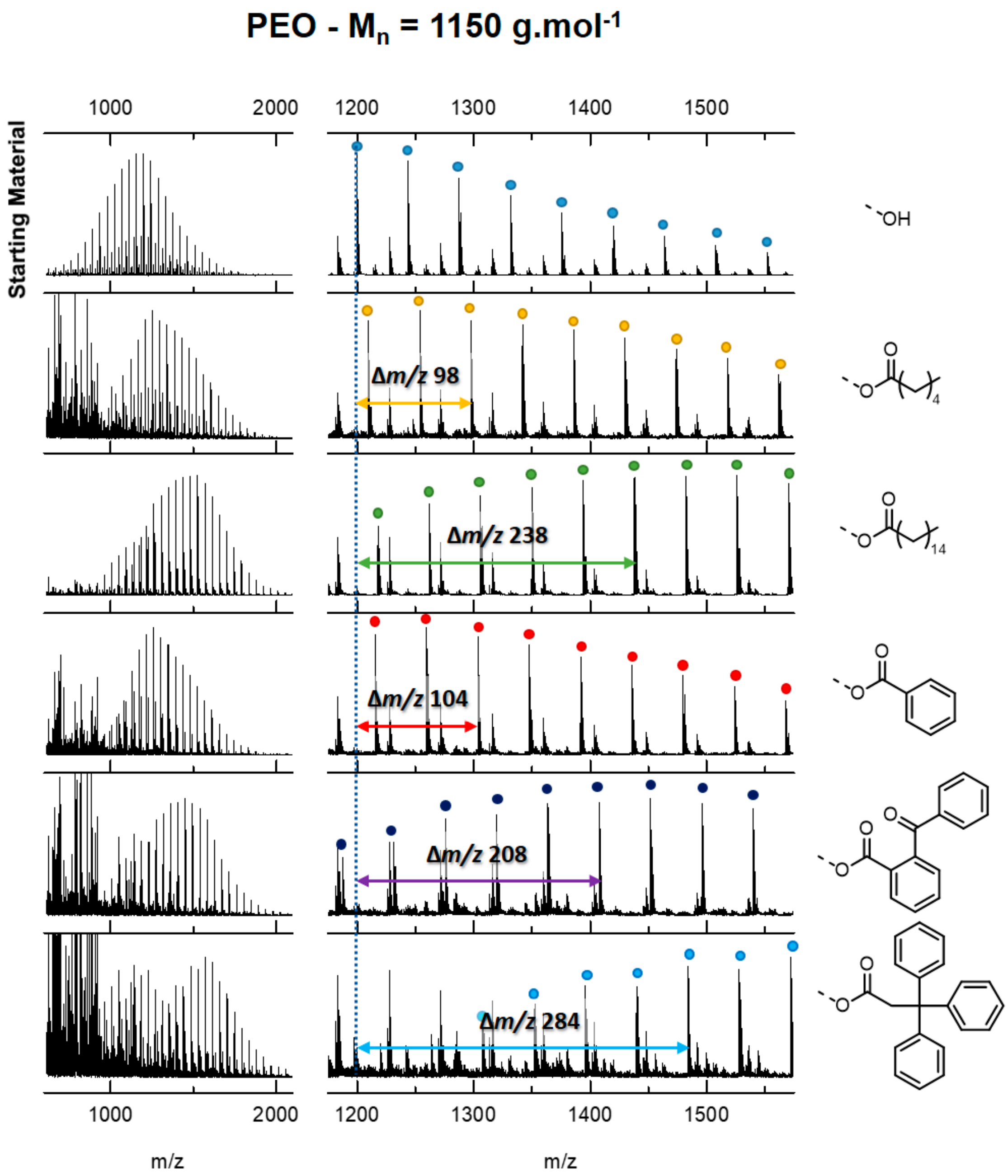

Figure SI2. MALDI mass spectra recorded for functionalized and non-functionalized PEO with M_n = 1150 g mol^{-1}. Functionalization was attested by apparition of additional signals with adequate mass differences compared to the α-methyl, ω-hydroxy polymer.

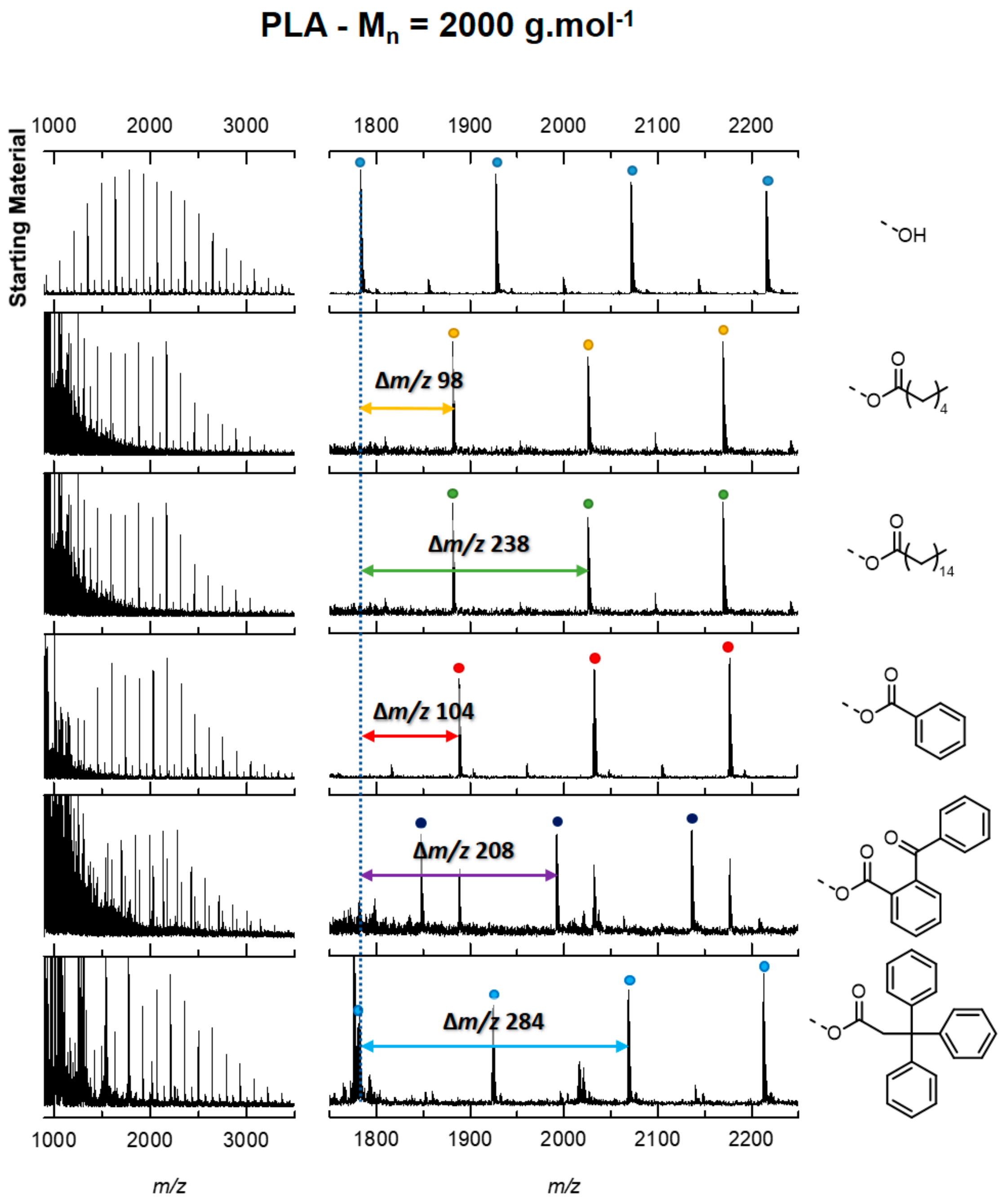

Figure SI3. MALDI mass spectra recorded for functionalized and non-functionalized PLA with M_n = 2000 g mol^{-1}. Functionalization was attested by apparition of additional signals with adequate mass differences compared to the α-methyl, ω-hydroxy polymer.

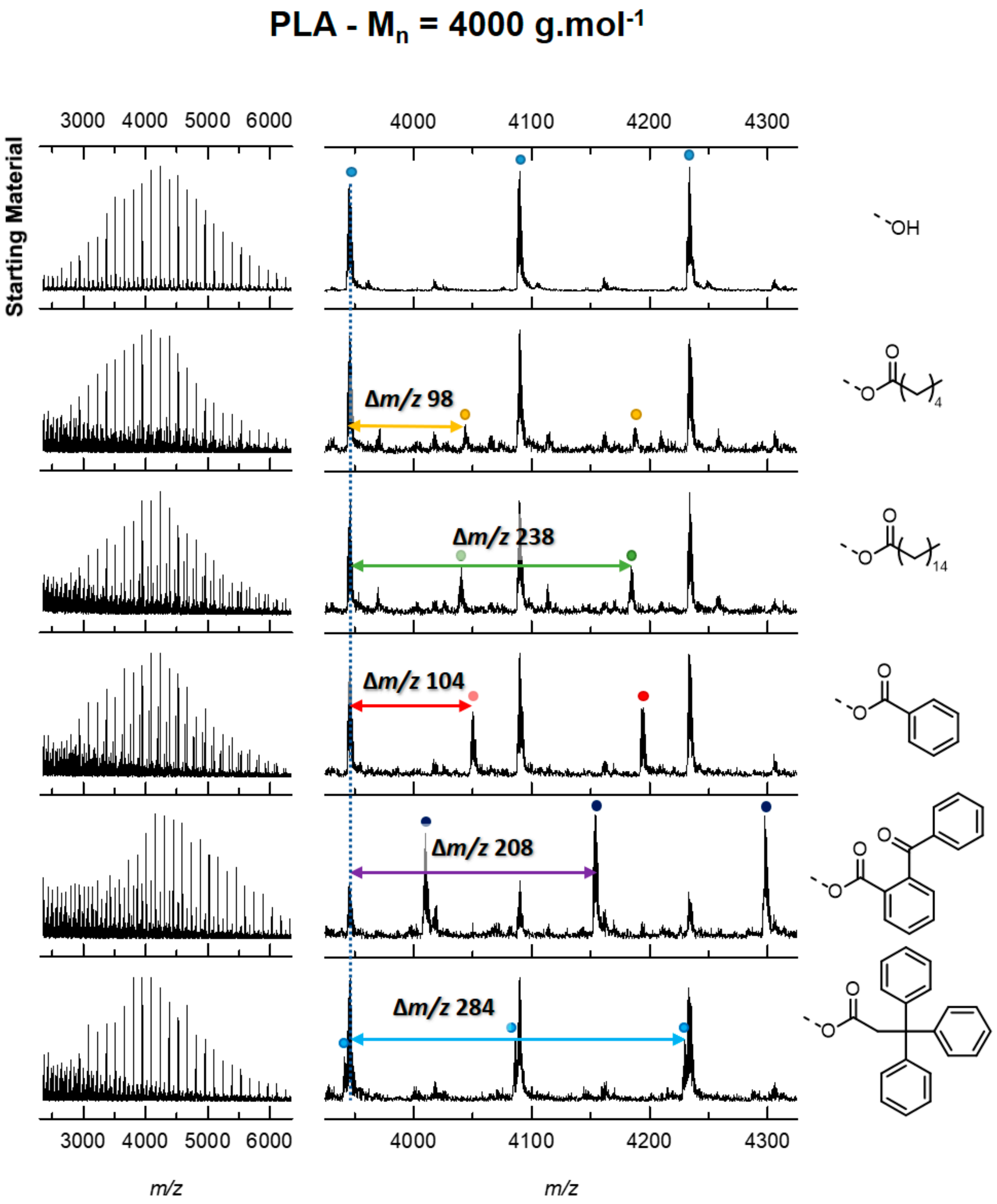

Figure SI4. MALDI mass spectra recorded for functionalized and non-functionalized PLA with M_n = 4000 g mol^{-1}. Functionalization was attested by apparition of additional signals with adequate mass differences compared to the α-methyl, ω-hydroxy polymer.

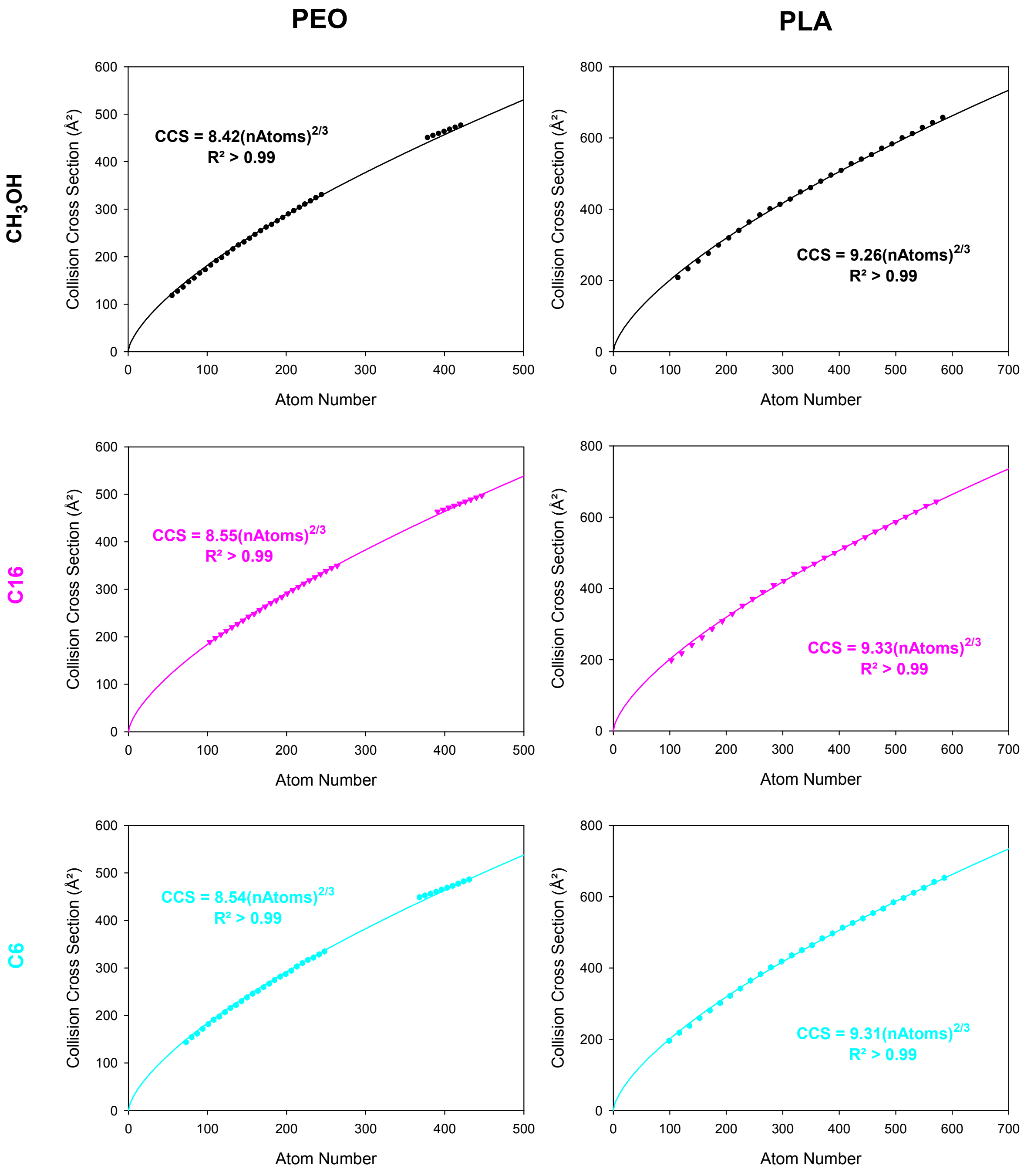

Figure SI5. Evolution of the collision cross section as a function of the number of atoms for singly and doubly charged globular ions for pristine and functionalized PEO and PLA with aliphatic end groups. Fittings were performed using the equation $CCS = A' \, nAtoms^{\frac{2}{3}}$; the equations and regression coefficients of each fit are shown.

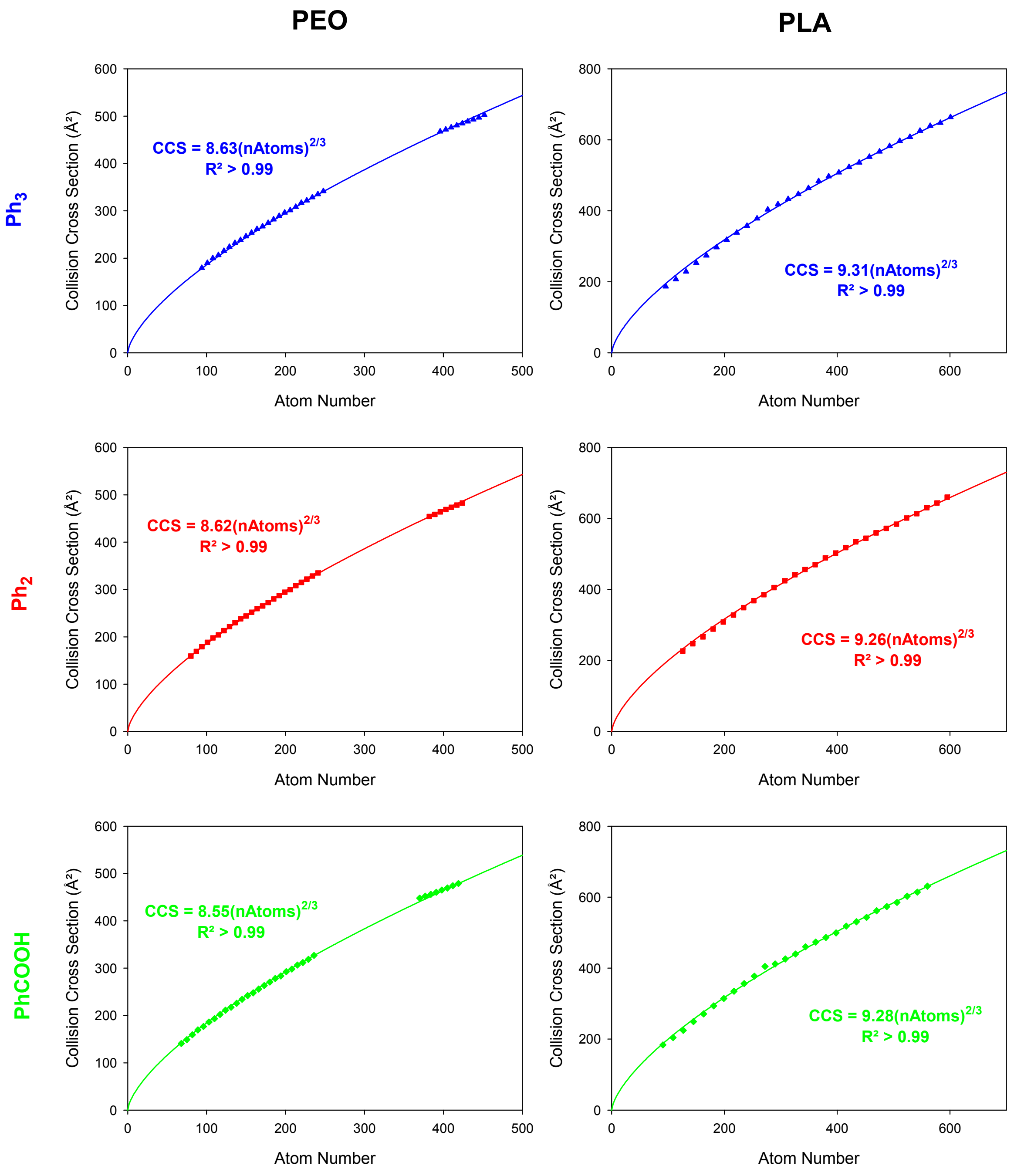

Figure SI6. Evolution of the collision cross section as a function of the number of atoms for singly and doubly charged globular ions for functionalized PEO and PLA with aromatic end groups. Fittings were performed using the equation $CCS = A'\ nAtoms^{\frac{2}{3}}$; the equations and regression coefficients of each fit are shown.
